# Beyond a bigger brain: Multivariable structural brain imaging and intelligence

**DOI:** 10.1016/j.intell.2015.05.001

**Published:** 2015

**Authors:** Stuart J. Ritchie, Tom Booth, Maria del C. Valdés Hernández, Janie Corley, Susana Muñoz Maniega, Alan J. Gow, Natalie A. Royle, Alison Pattie, Sherif Karama, John M. Starr, Mark E. Bastin, Joanna M. Wardlaw, Ian J. Deary

**Affiliations:** a Department of Psychology, The University of Edinburgh, United Kingdom; b Centre for Cognitive Ageing and Cognitive Epidemiology, The University of Edinburgh, United Kingdom; c Centre for Clinical Brain Sciences, The University of Edinburgh, United Kingdom; d Brain Research Imaging Centre, The University of Edinburgh, United Kingdom; e Scottish Imaging Network, a Platform for Scientific Excellence (SINAPSE), United Kingdom; f Department of Psychology, School of Life Sciences, Heriot-Watt University, United Kingdom; g Department of Neurology and Neurosurgery, McConnell Brain Imaging Center, Montreal Neurological Institute, McGill University, Canada; h Department of Psychiatry, Douglas Mental Health University Institute, McGill University, Canada; i Alzheimer Scotland Dementia Research Centre, The University of Edinburgh, United Kingdom

**Keywords:** *g*-factor, Intelligence, Brain, MRI, Structural equation modelling

## Abstract

People with larger brains tend to score higher on tests of general intelligence (*g*). It is unclear, however, how much variance in intelligence other brain measurements would account for if included together with brain volume in a multivariable model. We examined a large sample of individuals in their seventies (*n* = 672) who were administered a comprehensive cognitive test battery. Using structural equation modelling, we related six common magnetic resonance imaging-derived brain variables that represent normal and abnormal features—brain volume, cortical thickness, white matter structure, white matter hyperintensity load, iron deposits, and microbleeds—to *g* and to fluid intelligence. As expected, brain volume accounted for the largest portion of variance (~ 12%, depending on modelling choices). Adding the additional variables, especially cortical thickness (+~ 5%) and white matter hyperintensity load (+~ 2%), increased the predictive value of the model. Depending on modelling choices, all neuroimaging variables together accounted for 18–21% of the variance in intelligence. These results reveal which structural brain imaging measures relate to *g* over and above the largest contributor, total brain volume. They raise questions regarding which other neuroimaging measures might account for even more of the variance in intelligence.

## Highlights

• Brain size is known to correlate with general intelligence (*g*).
• It is unclear which other neuroimaging variables contribute beyond total brain size.
• We model multiple brain measures and *g* in a large sample aged around 73 years.
• All brain variables together account for around 20% of variance in *g*.

## 1 Introduction

Half or more of the variance in human intelligence test performance is accounted for by the general factor of cognitive ability, or *g* (Gignac & Watkins, 2013; Jensen, 1998). An extensive literature shows the importance of *g* for many educational, occupational, and health outcomes (Deary, 2012). Despite this, relatively little is known about the biological basis of *g* (see Deary, Penke, & Johnson, 2010, and Luders, Narr, Thompson, & Toga, 2009, for overviews). Here, we focus on what structural magnetic resonance imaging (MRI)-derived brain variables can contribute with respect to accounting for variance in *g*. We model several structural brain measures together to provide a reliable estimate of the association between brain structure and *g* at age 73.

The best-replicated neuroanatomical predictor of *g* is total brain volume (TBV; Galton, 1888; Gignac, Vernon, & Wickett, 2003; McDaniel, 2005; Pietschnig, Penke, Wicherts, Zeiler, & Voracek, 2014; Rushton & Ankney, 2009). Since TBV is associated with the overall number of neurons (e.g. Pakkenberg & Gundersen, 1997), it is plausible that larger brains allow for more complex, distributed cognitive processing. Initial brain imaging studies found widely-divergent estimates of the TBV-*g* correlation (Yeo, Turkheimer, Raz, & Bigler, 1987, found a correlation of *r* = .07, whereas Willerman, Shultz, Rutledge, & Bigler, 1991, found correlations as high as *r* = .51 in men). In a recent meta-analysis of 148 samples (total *N* = 8036, including the participants from the present study), Pietschnig et al. (2014) calculated an overall correlation between TBV and cognitive ability of *r* = .24 (~ 6% shared variance; note that some studies included observed and some included latent estimates of cognitive ability — the correlation may have been larger had all studies used latent *g*). In a sample of twins, Posthuma et al. (2002) showed that TBV and cognitive ability are genetically, as well as phenotypically, correlated.

A number of finer-grained MRI measures have also been associated with intelligence. For instance, measures of cortical thickness from regions across the brain have shown moderately-sized positive correlations with cognitive ability, potentially because they represent the density and arrangement of neurons in brain regions vital for cognition, such as prefrontal areas (e.g. Narr et al., 2007). Measures of the networks that support information transfer within the brain have also shown predictive validity for cognitive ability; in the same sample analyzed in the present study, a general factor of brain white matter tract structure measured by diffusion tensor MRI accounted for about 10% of the variance in *g* (Penke, Muñoz Maniega et al., 2012). Measures of damage to the white matter tracts, such as volume of white matter hyperintensities (WMH; Valdés Hernández et al., 2013), number of microbleeds (Cordonnier, Al-Shahi Salman, & Wardlaw, 2007; Werring et al., 2004), and number of iron deposits (Penke, Valdés Hernández et al., 2012) have also shown modest predictive validity for cognitive ability at age about 73. In a review of the literature on individual differences, Lubinski (2000) noted that the “biological phenomena [linked to *g*] are in no way mutually exclusive and can be complementary to one another” (p. 418). Thus, modelling these additional brain metrics alongside TBV will improve our understanding of whether they relate to general intelligence beyond brute brain size.

To our knowledge, no studies to date have included all of these structural brain variables together in a single model to assess their incremental predictive validity for intelligence. It is thus unclear whether they would each account for separate portions of variance, or whether the finer-grained variables would account for little after more global measures such as total brain volume are included. In the present study, we ask two questions. First, what is the best estimate of the percentage variance in *g* accounted for by the above brain measures when they are modelled together? Second, which brain imaging parameters have significant associations with *g* beyond total brain volume?

## 2 Method

### 2.1 Participants

Participants were members of the Lothian Birth Cohort 1936 (LBC1936; Deary et al., 2007; Deary, Gow, Pattie, & Starr, 2012), a sample of White European, community-dwelling older individuals. Most took part, at approximately 11 years of age, in the 1947 Scottish Mental Survey (Scottish Council for Research in Education, 1949). In Wave 1 of the LBC1936 study, 1091 of these individuals were followed-up when they were aged approximately 70 years old in 2004–07. In Wave 2, 886 (418 females) took part at age approximately 73 years, 700 of whom underwent brain MRI (Wardlaw et al., 2011). The data used in the present study come from Wave 2. Of the 700 who underwent brain MRI, 28 participants were removed from the current sample either based on quality control of the imaging data, or for scoring less than 24 on the Mini-Mental State Examination (Folstein, Folstein, & McHugh, 1975), a commonly-used screening instrument for possible dementia. A total of 672 individuals (319 female, 47.5%) therefore provided data for the present study. These individuals had an average age of 72.49 years (SD = 0.71) and had an average of 10.8 years of education (SD = 1.1 years). Written informed consent was obtained from all participants before their inclusion in the study, and the study was approved by the Multi-Centre Research Ethics Committee for Scotland (MREC/01/0/56) and the Lothian Research Ethics Committee (LREC/2003/2/29).

### 2.2 Measures

#### 2.2.1 Cognitive ability

The LBC1936 participants were administered fifteen cognitive tests at Wave 2. Three subtests were included from the Wechsler Memory Scale, 3rd Edition (WMS-III; Wechsler, 1998a): Logical Memory (immediate and delayed), Verbal Paired Associates (first and second recall), and Spatial Span (forwards and backwards). For this age group, the mean test–retest reliability coefficient of these subtests is .86 (Wechsler, 1998a). Six subtests were included from the UK version of the Wechsler Adult Intelligence Scale, 3rd Edition (WAIS-III^UK^; Wechsler, 1998b): Digit Symbol Coding, Digit Span Backwards, Block Design, Letter–Number Sequencing, Matrix Reasoning, and Symbol Search (mean test–retest reliability estimate = .83; Wechsler, 1998b). The participants completed the National Adult Reading Test (NART; Nelson & Willison, 1991) and the Wechsler Test of Adult Reading (WTAR; Holdnack, 2001). Both these reading tests have reliability coefficients > .87 (Kreutzer, DeLuca, & Caplan, 2011). A measure of verbal fluency (using the letters C, F, and L; Lezak, 2004; test–retest reliability = .74; Tombaugh, Kozak, & Rees, 1999) was administered. The participants completed three elementary cognitive assessments of processing speed: Simple Reaction Time, Choice Reaction Time (both described in detail by Deary, Der, & Ford, 2001), and visual Inspection Time (Deary et al., 2007). These three measures have estimated reliability coefficients of .62, .92, and .81, respectively (Deary, Johnson, & Starr, 2010).

Here, we assessed the extent to which MRI variables predict both overall *g*, and ‘fluid’ *g*. All of the above measures were included in the overall *g*-factor of intelligence. In the alternative models where a fluid *g* was calculated, the model included only the following subset of tests: Digit Symbol Coding, Digit Span Backwards, Block Design, Symbol Search, Letter–Number Sequencing, Matrix Reasoning, Simple and Choice Reaction Time and Inspection Time.

#### 2.2.2 Brain MRI

A full description of the image acquisition and the extraction of the different variables has been published elsewhere (Wardlaw et al., 2011; see Table 1 in that paper for MRI sequence details). Here, we provide a brief description for each variable. Many of the variables presented here have been individually investigated in relation to cognitive ability in the current sample. Interested readers should refer to Royle et al. (2013) for TBV, Valdés Hernández et al. (2013) for WMH load, Penke, Maniega et al. (2012) for the general factor of white matter tract structure (*g*FA), Karama et al. (2014) for cortical thickness, and Penke, Valdés Hernández et al. (2012) for iron deposits and microbleeds.

#### 2.2.3 Image acquisition

Participants underwent whole brain structural and high angular resolution 2 mm isotropic voxel diffusion MRI (7T2- and 64 diffusion-weighted (b = 1000 s/mm^2^) axial single-shot spin-echo echo-planar imaging volumes) on a GE Signa Horizon HDxt 1.5 T clinical scanner (General Electric, Milwaukee, USA) operating in research mode using a self-shielding gradient set (maximum gradient 33 mT/m), and an 8-channel phased-array head coil. Structural MRI included T2- (T2W), T2*-(T2*W) and FLAIR-weighted axial sequences, and a high resolution 3D T1-weighted volume scan. Analyses of MRI data described here were performed blind to all cognitive and clinical data, and structural images were analyzed seperately from DTI.

#### 2.2.4 Total brain volume & white matter hyperintensities

**Fig. 1:**
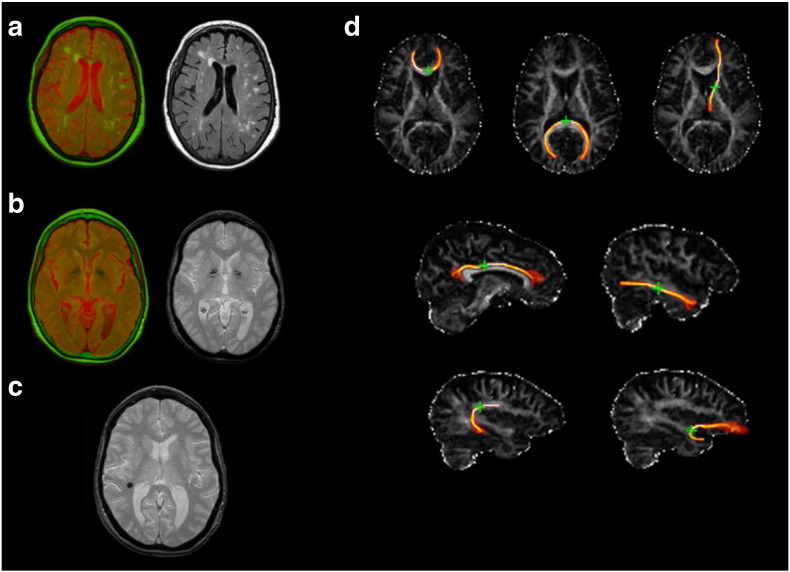
Example images of brain MRI features measured. (a) White matter hyperintensities (WMH): fused T2*W and FLAIR images mapped in red and green colour showing WMH in light green, left, and original FLAIR image, right. (b) Iron deposits: fused T2*W and FLAIR images mapped in red and green colour, showing iron deposits in dark green, left, and original T2*W image, right. (c) Microbleed (in black) as seen in T2*W. (d) FA maps with examples of segmented white matter tracts, with green cross indicating the seed point: genu and splenium of the corpus callosum and anterior thalamic radiation (upper row); rostral cingulum and inferior longitudinal fasciculus (middle row); arcuate and uncinate fasciculi (lower row).

TBV (cm^3^) was estimated by subtracting cerebrospinal fluid (CSF) from the intracranial volume (ICV). ICV was obtained semi-automatically using the T2*W sequence and the Object Extraction Tool in Analyze 9.0 (Mayo Clinic, Analyze 9.0. AnalyzeDirect, Inc. Mayo Clinic). ICV included the contents of the inner skull table with its inferior limit in the axial slice just superior to the tip of the odontoid peg at the foramen magnum and inferior to the inferior limits of the cerebellar tonsils (Wardlaw et al., 2011). CSF was extracted using MCMxxxVI ‘1936’ (Valdés Hernández, Ferguson, Chappell, & Wardlaw, 2010; Valdés Hernández et al., 2012), a semi-automatic multispectral segmentation technique with good inter-rater reliability that uses colour fusion to enhance tissue differentiation. T2*W and FLAIR sequences mapped in red and green colour space respectively were used to extract both CSF (red) and WMH (green). WMH were defined as punctate or diffuse +/− confluent areas in the white matter and deep grey matter, identified using the T2*W and FLAIR sequences as described above. All segmented images were visually checked for incorrectly classified tissue and manually corrected. For all analyses, we expressed WMH as a percentage of ICV. Fig. 1(a) provides an example of a brain image showing WMH.

#### 2.2.5 Fazekas ratings of WMH

As well as quantitative assessment of WMH volume, WMH burden was also assessed, based on the T2*W and FLAIR-weighted sequences using the Fazekas scale (Fazekas et al., 2002). Visual ratings were performed by an expert neurologist. WMH were coded in both the deep and periventricular white matter. Here we combine these scales using a simple sum of the ratings for the left and right hemisphere to yield two variables: Total Deep and Total Periventricular Fazekas score.

#### 2.2.6 Iron deposits

The MCMxxxVI technique was also used to assess iron deposits in the basal ganglia. This process is described in detail by Penke, Valdés Hernández et al. (2012). Briefly, affine co-registered T2*W and FLAIR volumes were fused in red/green colour space using FSL's FLIRT, and the brain was extracted using Analyze 9.0. The fused red/green volumes were then converted into a clustered sequence with 32 colour levels using minimum variance quantization. The clusters that correspond to iron deposits—shown in Fig. 1(b)—were then selected by mapping them in normalized red/green space and visually determining the range of green that best segmented these features. For the present analysis, the volume of iron deposits on the scan was rated by an expert neuroradiologist for each hemisphere on a scale of 1–4 (none/minimal/some/much), then the scores across the two hemispheres were summed, for a maximum score of 8.

#### 2.2.7 Microbleeds

Microbleeds were assessed by the neuroradiologist from the structural sequences using a modified version of the Brain Observer Microbleeds Scale (BOMBS; Cordonnier et al., 2009). Cordonnier et al. (2009) showed moderate-to-high interrater agreement using this scale. Here, we summed across the BOMBS ratings of different regions to form a total microbleeds variable for subsequent analyses. A brain image illustrating a microbleed is shown in Fig. 1(c).

#### 2.2.8 White matter tract segmentation

Diffusion MRI data were preprocessed using FSL tools (FMRIB, Oxford, UK; http://www.fmrib.ox.ac.uk). Connectivity data were generated using BedpostX/ProbTrackX, using default settings (Behrens et al., 2007). Twelve tracts were identified and extracted using probabilistic neighbourhood tractography implemented in the TractoR package for fibre tracking analysis (Clayden et al., 2011; http://www.tractor-mri.org.uk). This method of tractography has good reproducability (Clayden, Storkey, Maniega, & Bastin, 2009). The twelve segmented tracts were the Genu and Splenium of the Corpus Callosum; Bilateral Anterior Thalamic Radiations; Bilateral Rostral Cingulum Bundles; and the Bilateral Arcuate, Uncinate and Inferior Longitudinal Fasciculi. For illustrations of these tracts, see Fig. 1(d). Tract masks generated by probabilistic neighbourhood tractography were overlaid on the FA parametric maps and tract-averaged FA values, weighted by the connection probability, were determined for each tract in every subject. For subsequent analyses, we modelled a general factor of white matter tract structure (*g*FA) following Penke, Muñoz Maniega et al. (2012); see Section 2.3, below).

#### 2.2.9 Cortical thickness

To obtain local cortical thickness measurements for each subject, T1-weighted volume scans were processed by the CIVET pipeline (version 1.1.12; Kim et al., 2005) developed at the Montreal Neurological Institute (http://www.bic.mni.mcgill.ca). Steps (described in detail previously by Karama et al., 2009) include 1) linearly registering T1-weighted images to a standardized space using an age-specific template for the population under study; 2) correcting for intensity non-uniformity artifacts; 3) classifying the image into white matter (WM), grey matter (GM), cerebrospinal fluid (CSF), and background using a neural net classifier; 4) fitting images with a deformable mesh model to extract 2-dimensional inner (WM/GM interface) and outer (pial) cortical surfaces for each hemisphere and, through this process, producing high-resolution hemispheric surfaces with 81,924 polygons each (40,962 nodes or vertices per hemisphere); 5) registering both cortical surfaces for each hemisphere non-linearly to a high resolution average surface template in order to establish inter-subject correspondence of vertices; 6) applying a reverse of the linear transformation performed on the volume to allow vertex-based corticometric measurements in the native space of the magnetic resonance image; 7) calculating cortical thickness at each vertex; and 8) blurring each subject's cortical thickness map using a 20-millimeter full width at half maximum surface-based diffusion smoothing kernel (a necessary step to impose a normal distribution to corticometric data and to increase signal to noise ratio). In order to estimate vertex-based cortical volumes, prisms were extruded between the white and pial surfaces, in native space, and their volumes calculated by Gauss–Legendre quadrature. A third of the volume of each prism was then assigned to its respective three vertices forming the triangular base of the prism. The volume value for each vertex corresponded to the sum of all partial prism volumes linked to it. Total cortical thickness, the variable used in the models for the present study, was estimated by summing all vertex volume values across the cortex.

### 2.3 Statistical analysis

The structural equation modelling analyses were performed using MPlus version 7.2 (Muthén & Muthén, 1998–2014). As can be seen in Table S1, not all participants had data for all measures. As such, full information maximum likelihood estimation was used for all analyses. In addition, each variable was adjusted for sex, age (in days) at measurement, and handedness (hemispheric white matter tract measurements only) before it was included in the model, by obtaining the residuals after regressing it on these covariates.

A bi-factor model was used to provide a best estimate of the overall *g*-factor. Here, four specific group factors (nonverbal reasoning, speed, verbal declarative memory, and knowledge) were modelled along with a single general factor. The makeup of these group factors is described in full in the Supplemental Online Materials. The specific factors were uncorrelated with the general factor. Factors were identified by fixing variances to 1. Fluid *g* was estimated as a single latent factor from nine tests, marked with asterisks in Table S1. In the fluid models, four correlated residuals were included a priori to account for local dependencies hypothesized to be due to test format. These were between Digit Symbol and Symbol Search, Matrix Reasoning and Block Design, simple and choice reaction time, and Digit Span Backwards and Letter–Number Sequencing.

Total WMH load was measured using a single latent factor with three indicators, namely, WMH volume as a percentage of ICV, and total deep and periventricular Fazekas score (see Valdés Hernández et al., 2013). We also included a latent variable for general white matter tract structure (*g*FA; see Penke, Maniega et al., 2012). Here, correlated residuals were included between FA values for the same tract from the left and right hemisphere. Both latent variables were identified by fixing their variances to 1.

**Fig. 2:**
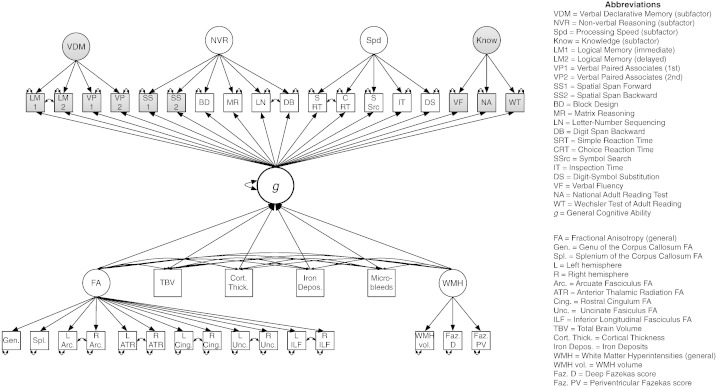
Path diagram of the full MIMIC model showing the bifactor model of cognitive ability, and general factors of fractional anisotropy and white matter lesions. This diagram depicts Model 1 (including overall *g* and TBV; see Table 3 in the main article). Shaded variables were not used in Models 2 or 4 (fluid *g*). Models 3 and 4 (‘cortical split’) replaced the TBV and Cortical Thickness variables with two manifest variables: Total Cortical and Total Subcortical tissue volumes.

We tested four MIMIC (multiple indicators, multiple causes) models, of the type used by Kievit et al. (2012), who showed that such models were the most appropriate with which to model brain–cognition relationships. The full model is shown in Fig. 2. MIMIC model 1 included TBV, cortical thickness, total WMH load, *g*FA, iron deposits, and microbleeds as determinants of a *g*-factor with all cognitive subtests as indicators. By inspecting this model, we were able to estimate the overall percentage of variance in *g* accounted for by the neuroanatomical variables. Kievit et al. (2012) also tested several other model classes. We tested three alternatives to our MIMIC models: simple ‘neuro-*g*’ models, correlation models, and a reversed version of the MIMIC model shown here. These alternatives to the main MIMIC model are described in Fig. S2 and Table S5 in the Supplemental Materials.

Three more MIMIC models, each of which was similar to MIMIC model 1, were estimated. MIMIC model 2 used the same brain measures, but restricted the *g*-factor indicators to the fluid tests listed above. This allowed us to test skills that are known to decline strongly with age (Salthouse, 2004), and which might thus have had a stronger relation to the brain variables in our older-age sample. MIMIC model 3 dropped the variables of TBV and cortical thickness and instead used cortical and subcortical tissue volumes as separate variables in a model with an overall *g*-factor, in order to note the relative contribution of the volume of these types of tissue in accounting for variance in cognitive ability. MIMIC model 4 used the same brain variables as Model 3 with the fluid *g*-factor from Model 2.

Model fit was assessed based on a number of fit indices with commonly-accepted cutoffs from the published literature. Specifically we focused on the comparative fit index (CFI), the Tucker–Lewis index (TLI), the root mean square error of approximation (RMSEA) and the standardized root mean square residual (SRMR). We adopted cut-offs of ≥ 0.95 for the CFI and TLI, ≤ 0.08 for the RMSEA and ≤ 0.06 for the SRMR as being indicative of good model fit.

As a final analysis, we also explored whether the regression coefficients of interest differed between male and female cohort members, using multi-group SEM. Using the four MIMIC models described above, we first established metric measurement invariance for the latent constructs across males and females. Next, we placed equivalence constraints on each of the parameters of interest and explored whether this resulted in a significant loss of model fit based on a χ^2^ difference test. For these analyses we used data residualized only for participants' age and handedness (where appropriate).

## 3 Results

Descriptive statistics for all brain and cognitive test variables are provided in the Supplementary Materials (Table S1). The correlation matrix in Table 1 shows how each of the brain variables correlated with one another and with the overall *g*- and fluid *g*-factors. Tables S2, S3, and S4 in the Supplementary Materials show the loadings of each of the cognitive and brain measures on the factors in each of the models. As would be expected, cortical and subcortical volumes had large positive correlations with TBV (*r* = .81 and .95, respectively). Similarly, overall and fluid *g* correlated highly (*r* = .87). Both overall and fluid *g* had correlations of small-to-moderate size with each of the brain imaging variables (*r* = ± .07 to .32 and *r* = ± .09 to .32, respectively).

**Fig. 3:**
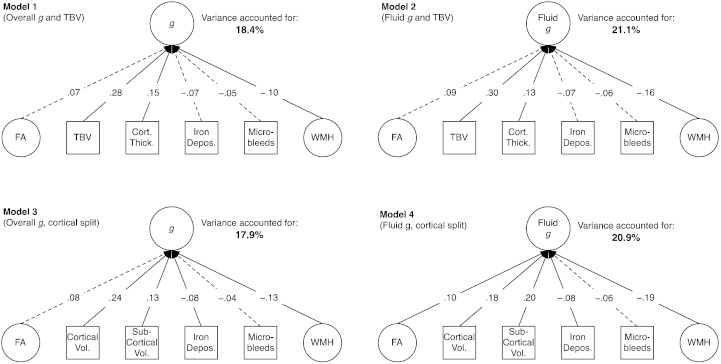
Simplified path diagrams of Models 1–4, showing the percentage variance in general intelligence (*g*) or general fluid intelligence (Fluid *g*) accounted for by each of the structural neuroimaging parameters. Values on each path are standardized coefficients. Dotted lines indicate paths that were not statistically significant (see Table 3 for full details). Full model (including all indicators of latent variables) shown in Fig. 2. Abbreviations: FA = general Fractional Anisotropy; TBV = Total Brain Volume; Cort. Thick. = Cortical Thickness; Depos. = Deposits; WMH = general White Matter Hyperintensities; Vol. = Volume.

All four MIMIC models had acceptable fit to the data, as shown in Table 2. The standardized parameter estimates for the imaging variables in each of the models are shown in Table 3. In MIMIC model 1, which included the overall *g*-factor and TBV, 18.4% of the variance in *g* was shared with the brain variables (this percentage was 21.1%, 17.9%, and 20.9% in MIMIC models 2, 3, and 4, respectively). Thus, the brain variables tended to account for more substantial percentages of variance in fluid than overall *g* (models 2 & 4 versus models 1 & 3), and using cortical and subcortical tissue volumes in place of TBV tended to slightly reduce the variance accounted for (models 1 & 2 versus models 3 & 4). Generally, however, the shared variances remained similar regardless of these modelling choices. The regression coefficients of each variable did not change substantially, although in some cases the effect (for instance, for iron deposits) was below the threshold for statistical significance. Fig. 3 illustrates each of the models in simplified form.

As a final robustness check, we residualised TBV for ICV, to adjust for any age-related shrinkage of the brain (Aribisala et al., 2013). This had the effect of reducing somewhat the variance accounted for in overall *g* to 14.6% (in Model 1) and increasing somewhat the variance accounted for in fluid *g* to 24.0% (in Model 2).

The fit statistics for the alternatives-to-MIMIC models (simple, correlational, and reversed; diagrams shown in Fig. S1), are provided in Table S5 in the Supplementary Materials. In all cases, these models had poorer fit than our main MIMIC model (all sample-adjusted Bayesian Information Criterion values were higher than the equivalent main model). The reverse MIMIC models were closer in fit than the simple or correlational models to the main models, and themselves all had acceptable or near-to-acceptable fit. These alternative models were thus also viable representations of the data. Importantly, however, all the models produced broadly similar values for shared variance between neuroimaging parameters and general cognitive ability (17.9% to 25.2%, depending on modelling choices).

Finally, we explored whether the observed coefficients were equivalent across male and female participants. We found no evidence for sex differences in either the measurement of the latent variables, or in the magnitudes of the relationships between the brain imaging metrics and measures of cognitive ability. Full results are provided in Supplementary Material Tables S6 and S7.

## 4 Discussion

In a large sample of older adults, we showed that a selection of brain imaging measurements account for about 20% of the variance in a latent trait of general cognitive ability. The highest percentages of variance accounted for were found in models using a ‘fluid’ factor of intelligence, but the percentages for a comprehensive, fifteen-test general intelligence factor were similar. The percentages were broadly comparable whether or not TBV was split into cortical and subcortical components. Given the large sample size, the wide range of brain and cognitive variables, and multivariate modelling approach, these results represent one of the most accurate figures to date on the percentage variance in intelligence accounted for by structural brain imaging features. Our model integrates many of the variables examined thus far in ‘vertical’ investigations of *g* (that is, reductionistic investigations of the causes of variation in *g*; Jensen, 1998, p. 578; Lubinski, 2000; or what Deary, 2000, called “looking down on human intelligence”).

In MIMIC model 1, with an overall *g*-factor and TBV included, the contributions of *g*FA, iron deposits, or microbleeds were not significant beyond the effects of TBV, cortical thickness, and WMH load. These variables did share a small amount of variance with cognitive ability, but not a large enough amount to be statistically significant in our sample. This is not to say that the variables found to be non-significant in this sample are not contributors to intelligence: In populations with, for instance, more damage to their white matter tracts, these variables might account for different proportions of the variance. From a cognitive ageing perspective, however, it is of interest that in three of the four models, *g*FA made no significant contribution to explaining cognitive variance in the multivariate model, whereas white matter hyperintensities were always a significant predictor. This is consistent with previous multivariate studies where hyperintensities have been the strongest predictor of cognitive functioning, with FA reduced to non-significance (e.g. Meier et al., 2012).

Each of the measures found to be significant in our models has a plausible mechanistic basis for its link to cognitive ability. As noted above, TBV and cortical thickness have been related to the number, density, and arrangement of neurons (Pakkenberg & Gundersen, 1997; Shaw et al., 2006). WMH are theorized to disrupt efficient information processing via the white matter tracts (Schmidt et al., 1993); our finding that WMH load accounted for more variation in fluid than in overall *g* is consistent with this view, since fluid *g* relied more strongly on speeded tests. FA has been shown to have substantial relations to cognitive processing speed (e.g. Turken et al., 2008), indicating that measures of white matter tract water diffusion anisotropy can be used to index information processing efficiency.

Whereas 18–21% of the variance in cognitive ability accounted for by brain measures is substantial (and likely reflects a lower-bound estimate due to the necessarily imperfect measurement of each brain parameter), the present findings raise the question of how we can account for an even larger portion of the variance. A number of structural brain parameters were not measured in the LBC1936 sample, for example cortical convolution (e.g. Luders et al., 2008) and callosal thickness (e.g. Luders et al., 2011), though they could potentially be derived in future. It is plausible that these would account for extra variance if they were included in the model. Analysis from different perspectives may also improve the predictive validity of brain-intelligence models, including a fine-grained ‘connectomics’ analysis (see, e.g. Griffa, Baumann, Thiran, & Hagmann, 2013), or an analysis at the even lower level of differences in the morphology of synaptic spines (Morrison & Baxter, 2014). The inclusion of functional neuroimaging measures (see Jung & Haier, 2007 for a review) may also allow for more accurate predictions of cognitive ability.

The strengths of this study lie in the large sample, the extensive range of both brain measures and cognitive tests, and our modelling approach that allowed us to go beyond basic correlation and regression designs by using error-free latent variables (although it should be noted that not all our variables were latent; the manifest variables that contributed to accounting for *g* are thus likely to be somewhat less reliable). In addition, the relatively narrow age, ability, and socioeconomic bracket of the cohort diminishes any confounding effects of variation in these factors.

Of course, the narrow age range also means that the present study's results apply to people aged about 73, and additional studies would be needed to test our models' generalizability to groups at other stages in the life course. Relatedly, some of the brain measures included here (for instance, WMH) are measures of accumulated damage that, by definition, is more commonly present in older individuals. Other brain measures may also show effects of age — for instance, the FA values are lower than they would be in younger people (e.g. Kochunov et al., 2012), reflecting age-related deterioration in white matter structure. Therefore, our results, based as they are on a mixture of normal, abnormal, and age-affected features of the brain, will be less applicable to younger samples where, for example, there are unlikely to be WMH, and mineral deposits will be less than at age 73. This explanation is congruent with our finding that, after taking into account brain atrophy (by correcting TBV for ICV), we found that a large proportion of variance in fluid intelligence—which more is more strongly affected by the ageing process (Salthouse, 2004)—was accounted for. Different measures may be required to capture similar proportions of variance in younger brains.

In the early 20th century, Pearson (1906) reanalyzed data from Galton (1888), and estimated that around 1% of the variance in educational attainment (a proxy for intelligence) was accounted for by head size (a proxy for brain size). Pearson argued that “the correlation is so small that it would be absolutely idle to endeavour to predict the intellectual ability of an individual from his or her head measurements” (p. 105). However, with the advent of neuroimaging methods, progress has been made: The present study showed that, modelled together, multimodal structural brain imaging measures, including but not limited to total brain volume, account for some 20% of the variance in general cognitive ability. Our results should act as a spur for future ‘vertical’ research on intelligence and the brain, since they raise the challenging question of which additional structural and functional neural variables might account for portions of the remaining variance in this key psychological trait.

## Author contributions

SJR, TB and IJD developed the study concept. Funding was obtained for the study by JMS, JMW, and IJD. Cognitive data were collected by JC, AJG, and AP. Brain imaging data were processed for analysis by MCVH, SMM, NAR, SK, and MEB. TB and SJR analyzed the data. SJR drafted the manuscript, and TB, JC, SMM, AJG, SK, JMS, MEB, JMW, and IJD provided critical additions and revisions.

## Figures and Tables

**Table 1:** Pearson correlation matrix for brain measures and both *g* measures (valid *n* range = 625 to 672).

Variable	1.	2.	3.	4.	5.	6.	7.	8.	9.
1. Total Brain Volume	–								
2. Cortical thickness	.22⁎⁎⁎	–							
3. Cortical tissue volume	.81⁎⁎⁎	.56⁎⁎⁎	–						
4. Subcortical tissue volume	.95⁎⁎⁎	.00	.59⁎⁎⁎	–					
5. Total WMH	− .03	− .25⁎⁎⁎	− .06	.04	–				
6. *g*FA	.19⁎⁎⁎	.34⁎⁎⁎	.27⁎⁎⁎	.10⁎	− .44⁎⁎⁎	–			
7. Iron deposits (basal ganglia)	− .03	− .11⁎⁎	− .07	.01	.02	.05	–		
8. Micro-bleeds	− .02	− .05	− .04	− .01	.09⁎	.02	.04	–	
9. Overall *g*	.31⁎⁎⁎	.24⁎⁎⁎	.32⁎⁎⁎	.24⁎⁎⁎	− .20⁎⁎⁎	.26⁎⁎⁎	− .09⁎	− .07	–
10. Fluid *g*	.32⁎⁎⁎	.23⁎⁎⁎	.30⁎⁎⁎	.27⁎⁎⁎	− .22⁎⁎⁎	.24⁎⁎⁎	− .08⁎	− .09⁎	.87⁎⁎⁎

Note: These correlations are based on factor scores from the measurement models described above for Total WMH (white matter hyperintensities), *g*FA (general fractional anisotropy), overall *g* and fluid *g*. Factor scores were computed using the regression method within MPlus. Factor determinacies from the complete data pattern, which range from 0.00–1.00 and provide a metric for the reliability of the factor scores, were .96, .92, .90 and .91 respectively. These values suggest the factor scores were reasonable estimates of the latent traits from the full structural equation models reported in the main analysis.

⁎⁎⁎ *p* < .001.

⁎⁎ *p* < .01.

⁎ *p* < .05.

**Table 2:** MIMIC model fit indices (N = 672).

	*χ^2^*	df	*p*-Value	CFI	TLI	RMSEA	SRMR	saBIC
MIMIC model 1: Overall *g* TBV	1024.01	586	< .001	0.951	0.945	0.033	0.043	59,149.41
MIMIC model 2: Fluid *g* TBV	576.58	324	< .001	0.952	0.945	0.034	0.041	45,434.25
MIMIC model 3: Overall *g* Cortical Split	1043.69	586	< .001	0.948	0.942	0.034	0.044	58,852.96
MIMIC model 4: Fluid *g* Cortical Split	588.12	324	< .001	0.949	0.941	0.035	0.042	45,139.20

Note: CFI = Comparative Fit Index; TLI = Tucker–Lewis Index; RMSEA = Root Mean Square Error of Approximation; SRMR = Standardized Root Mean Square Residual; saBIC = sample-adjusted Bayesian Information Criterion. TBV = Total Brain Volume.

**Table 3:** Standardized regression betas, confidence intervals, and incremental variance in intelligence explained by each of the neuroimaging variables in Models 1 to 4 (*n* = 672).

Standardized effect [95% CI]	Overall *g* Model 1		Fluid *g* Model 2		Overall *g* Model 3		Fluid *g* Model 4	
	*β* [95%CI]	Incremental variance	*β* [95%CI]	Incremental variance	*β* [95%CI]	Incremental variance	*β* [95%CI]	Incremental variance
Total Brain Volume	.28⁎⁎⁎ *[.20, .37]*	11.3% (+ 11.3%)	.30⁎⁎⁎ *[.22, .38]*	12.3% (+ 12.3%)	–	–	–	–
Cortical thickness	.15⁎⁎ *[.06, .25]*	16.0% (+ 4.7%)	.13⁎ *[.03, .22]*	17.3% (+ 5.0%)	–	–	–	–
Cortical tissue volume	–	–	–	–	0.24⁎⁎⁎ *[.14, .34]*	13.4% (+ 13.4%)	.18⁎⁎⁎ *[.09, .28]*	13.4% (+ 13.4%)
Sub-cortical tissue volume	–	–	–	–	0.13⁎ *[.03, .23]*	14.1% (+ .7%)	.20⁎⁎⁎ *[.10, .30]*	15.0% (+ 1.6%)
General white matter hyperintensities	− .10⁎ *[− .20, − .01]*	17.3% (+ 1.3%)	− .16⁎⁎ *[− .25, − .06]*	20.1% (+ 2.8%)	− .13⁎⁎ *[− .23, − .04]*	16.7% (+ 2.6%)	− .19⁎⁎⁎ *[− .28, − .09]*	19.9% (+ 4.9%)
General fractional anisotropy	.07 *[− .03, .17]*	17.5% (+ 0.2%)	.09 *[− .02, .19]*	20.3% (+ 0.2%)	.08 *[− .02, .18]*	16.9% (+ 0.2%)	.10⁎ *[.00, .20]*	20.1% (+ 0.2%)
Iron deposits (basal ganglia)	− .07 *[− .15, .00]*	18.1% (+ 0.6%)	− .07 *[− .14, .01]*	20.8% (+ 0.5%)	− .08⁎ *[− .16, − .01]*	17.7% (+ 0.8%)	− .08⁎ *[− .15, .00]*	20.7% (+ 0.6%)
Micro-bleeds	− .05 *[− .12, .02]*	18.4% (+ 0.3%)	− .06 *[− .14, .02]*	21.1% (+ 0.3%)	− .04 *[− .11, .03]*	17.9% (+ 0.2%)	− .06 *[− .13, .01]*	20.9% (0.2%)
Total variance accounted for	18.4% *p < .001*		21.1% *p < .001*		17.9% *p < .001*		20.9% *p < .001*	

Note: Model 1 = overall *g* with Total Brain Volume; Model 2 = fluid *g* with cortical split; Model 3 = overall *g* with Total Brain Volume; Model 4 = fluid *g* with cortical split. Predictors were entered into the model in the order in which they appear in the table.

⁎ *p* < .05.

⁎⁎ *p* < .01.

⁎⁎⁎ *p* < .001.

## Supplementary data

Supplementary Tables S1-S7, Supplementary Figure S1, and sex difference analysis.
