# Supplementary material for: Beyond a bigger brain: Multivariable structural brain imaging and intelligence
Source: Intelligence. 2015 Jul-Aug;51:47–56. doi: 10.1016/j.intell.2015.05.001 (PMC4518535; doi:10.1016/j.intell.2015.05.001)
Supplement: Supplementary file 1 — Supplementary Tables S1-S7, Supplementary Figure S1, and sex difference analysis. [file mmc1.docx]

Supplemental Online Materials for:

**Beyond a bigger brain: Multivariable structural brain imaging and intelligence**

Stuart J. Ritchie, Tom Booth, Maria del C. Valdés Hernández, Janie Corley, Susana Muñoz Maniega, Alan J. Gow, Natalie A. Royle, Alison Pattie, Sherif Karama, John M. Starr, Mark E. Bastin, Joanna M. Wardlaw, and Ian J. Deary

*Table S1:* Descriptive statistics for all cognitive tests and brain imaging measures.

|  | *n* | Mean | SD | Skew | Kurtosis |
| --- | --- | --- | --- | --- | --- |
| Logical Memory (Immediate) | 671 | 45.79 | 10.27 | *−*0.43 | 0.25 |
| Logical Memory (Delayed) | 671 | 28.88 | 8.06 | *−*0.52 | 0.20 |
| Verbal Paired Associates 1^st^ Recall | 662 | 2.80 | 2.30 | 0.62 | *−*0.69 |
| Verbal Paired Associates 2^nd^ Recall | 659 | 6.37 | 2.10 | *−*1.25 | 0.56 |
| Spatial Span Forward | 671 | 7.64 | 1.63 | *−*0.10 | *−*0.42 |
| Spatial Span Backwards | 670 | 7.11 | 1.61 | *−*0.06 | *−*0.14 |
| *Block Design | 670 | 34.29 | 10.00 | 0.46 | 0.09 |
| *Matrix Reasoning | 671 | 13.47 | 4.88 | *−*0.10 | *−*0.94 |
| *Letter-Number Sequencing | 672 | 11.00 | 3.00 | 0.30 | 0.39 |
| *Digit Span Backwards | 672 | 7.87 | 2.30 | 0.29 | *−*0.19 |
| *Simple Reaction Time | 672 | 0.27 | 0.05 | 1.67 | 4.12 |
| *Choice reaction Time | 672 | 0.65 | 0.09 | 0.89 | 1.71 |
| *Symbol Search | 671 | 24.77 | 6.10 | *−*0.32 | 0.75 |
| *Inspection Time Total | 660 | 111.39 | 11.80 | *−*1.35 | 5.03 |
| *Digit Symbol | 671 | 56.39 | 12.21 | 0.10 | *−*0.21 |
| Verbal Fluency Total | 671 | 43.31 | 12.77 | 0.23 | 0.11 |
| National Adult Reading Test | 671 | 34.60 | 7.90 | *−*0.53 | *−*0.09 |
| Wechsler Test of Adult Reading | 671 | 41.28 | 6.70 | *−*0.95 | 0.65 |
|  |  |  |  |  |  |
| *FA Tractography* |  |  |  |  |  |
| Genu of corpus callosum | 630 | 0.41 | 0.05 | *−*0.07 | *−*0.14 |
| Splenium of corpus callosum | 646 | 0.49 | 0.07 | *−*0.31 | 0.57 |
| L Arcuate Fasciculus | 622 | 0.45 | 0.04 | *−*0.42 | 0.44 |
| R Arcuate Fasciculus | 565 | 0.43 | 0.04 | *−*0.30 | 0.72 |
| L Anterior Thalamic Radiation | 541 | 0.32 | 0.03 | *−*0.11 | 0.24 |
| R Anterior Thalamic Radiation | 626 | 0.33 | 0.03 | *−*0.29 | 0.53 |
| L Rostral Cingulum | 624 | 0.44 | 0.05 | *−*0.54 | 0.79 |
| R Rostral Cingulum | 633 | 0.39 | 0.04 | *−*0.61 | 1.76 |
| L Uncinate Fasciculus | 552 | 0.33 | 0.03 | *−*0.12 | 0.39 |
| R Uncinate Fasciculus | 612 | 0.33 | 0.03 | *−*0.25 | 0.48 |
| L Inf. Longitudinal Fasciculus | 647 | 0.40 | 0.05 | *−*0.28 | 0.04 |
| R Inf. Longitudinal Fasciculus | 647 | 0.38 | 0.05 | *−*0.40 | 0.20 |
|  |  |  |  |  |  |
| *White Matter Hyperintensity* |  |  |  |  |  |
| White Matter Hyperintensity volume in ICV | 670 | 0.83 | 0.91 | 2.48 | 9.97 |
| Fazekas Deep Total | 672 | 1.09 | 0.67 | 0.49 | 0.67 |
| Fazekas Periventricular Total | 672 | 1.36 | 0.65 | 0.91 | 0.56 |
|  |  |  |  |  |  |
| *Other Neuroimaging Measures* |  |  |  |  |  |
| Total Brain Volume (cm^3^) | 670 | 1123.93 | 107.19 | 0.22 | *−*0.05 |
| Mean Cortical Thickness (mm) | 626 | 2.83 | 0.14 | *−*0.34 | 0.20 |
| Cortical Tissue Volume (cm^3^) | 626 | 451.32 | 40.57 | 0.12 | 0.02 |
| Subcortical Tissue Volume (cm^3^) | 625 | 672.64 | 74.37 | 0.25 | 0.13 |
| Iron Deposits (Basal Ganglia) rating | 672 | 3.47 | 1.71 | 0.79 | *−*0.54 |
| *n* Micro-bleeds | 672 | 0.23 | 1.13 | 11.98 | 189.17 |

Note: Mean age = 72.49 years (SD = 0.71); 353 male 319 female; L = left hemisphere; R = right hemisphere; *indicators of ‘fluid’ *g.*

*Table S2*. Factor loadings for cognitive tests in Model 1 (overall *g* with Total Brain Volume; see Figure 2 for a diagram and Table 3 for fit statistics). Loadings were very similar for Model 3, which also included overall *g*. For loadings on ‘fluid’ *g*, see Table S3.

| Factor | Cognitive test | Standardized loading | SE | *p* |
| --- | --- | --- | --- | --- |
| General cognitive ability (Overall *g*) | Logical Memory (Immediate) | .485 | .036 | <.001 |
|  | Logical Memory (Delayed) | .500 | .034 | <.001 |
|  | Verbal Paired Associates 1^st^ Recall | .394 | .037 | <.001 |
|  | Verbal Paired Associates 2^nd^ Recall | .477 | .037 | <.001 |
|  | Spatial Span Forward | .344 | .046 | <.001 |
|  | Spatial Span Backwards | .409 | .042 | <.001 |
|  | Block Design | .625 | .033 | <.001 |
|  | Matrix Reasoning | .605 | .032 | <.001 |
|  | Letter-Number Sequencing | .620 | .030 | <.001 |
|  | Digit Span Backwards | .514 | .035 | <.001 |
|  | Simple Reaction Time | −.283 | .043 | <.001 |
|  | Choice reaction Time | −.453 | .043 | <.001 |
|  | Symbol Search | .607 | .033 | <.001 |
|  | Inspection Time Total | .410 | .040 | <.001 |
|  | Digit Symbol | .663 | .030 | <.001 |
|  | Verbal Fluency Total | .515 | .039 | <.001 |
|  | National Adult Reading Test | .640 | .032 | <.001 |
|  | Wechsler Test of Adult Reading | .646 | .031 | <.001 |
| Verbal declarative memory (subfactor) | Logical Memory (Immediate) | .345 | .047 | <.001 |
|  | Logical Memory (Delayed) | .373 | .043 | <.001 |
|  | Verbal Paired Associates 1^st^ Recall | .544 | .050 | <.001 |
|  | Verbal Paired Associates 2^nd^ Recall | .694 | .057 | <.001 |
| Nonverbal reasoning (subfactor) | Spatial Span Forward | .544 | .083 | <.001 |
|  | Spatial Span Backwards | .523 | .074 | <.001 |
|  | Block Design | .223 | .067 | .001 |
|  | Matrix Reasoning | .156 | .068 | .022 |
|  | Letter-Number Sequencing | .144 | .048 | .003 |
|  | Digit Span Backwards | .117 | .054 | .029 |
| Speed (subfactor) | Simple Reaction Time | −.170 | .064 | .007 |
|  | Choice reaction Time | −.500 | .053 | <.001 |
|  | Symbol Search | .414 | .045 | <.001 |
|  | Inspection Time Total | .280 | .055 | <.001 |
|  | Digit Symbol | .509 | .049 | <.001 |
| Knowledge (subfactor) | Verbal Fluency Total | .112 | .044 | .011 |
|  | National Adult Reading Test | .705 | .071 | <.001 |
|  | Wechsler Test of Adult Reading | .686 | .072 | <.001 |

Note: Simple and Choice Reaction Time load negatively on their factors since lower scores indicate better (faster) performance.

*Table S3.* Factor loadings for cognitive tests in Model 2 (fluid *g* with Total Brain Volume; see Table 3 in the main article for fit statistics). Loadings were very similar for Model 4, the other model including fluid *g*.

| Factor | Cognitive test | Standardized loading | SE | *p* |
| --- | --- | --- | --- | --- |
| General fluid intelligence (Fluid *g*) | Block Design | .494 | .035 | <.001 |
|  | Matrix Reasoning | .420 | .033 | <.001 |
|  | Letter-Number Sequencing | .483 | .036 | <.001 |
|  | Digit Span Backwards | .372 | .037 | <.001 |
|  | Simple Reaction Time | −.297 | .041 | <.001 |
|  | Choice reaction Time | −.571 | .041 | <.001 |
|  | Symbol Search | .642 | .039 | <.001 |
|  | Inspection Time Total | .450 | .043 | <.001 |
|  | Digit Symbol | .716 | .031 | <.001 |

*Table S4*. Factor loadings for general Fractional Anisotropy (FA) and general White Matter Hyperintensities (WMH). Loadings shown for Model 1 (see Figure 2 and Table 3 in the main article); loadings were highly similar across all four models. For relations of these factors and and the other neuroimaging variables to *g*, see Table 4 in the main article.

| Factor | Neuroimaging variable | Standardized  loading | SE | *p* |
| --- | --- | --- | --- | --- |
| General FA | Genu of corpus callosum | .610 | .031 | <.001 |
|  | Splenium of corpus callosum | .334 | .043 | <.001 |
|  | L Arcuate Fasciculus | .620 | .034 | <.001 |
|  | R Arcuate Fasciculus | .613 | .033 | <.001 |
|  | L Anterior Thalamic Radiation | .643 | .030 | <.001 |
|  | R Anterior Thalamic Radiation | .663 | .030 | <.001 |
|  | L Rostral Cingulum | .605 | .037 | <.001 |
|  | R Rostral Cingulum | .564 | .040 | <.001 |
|  | L Uncinate Fasciculus | .682 | .031 | <.001 |
|  | R Uncinate Fasciculus | .669 | .028 | <.001 |
|  | L Inf. Longitudinal Fasciculus | .534 | .036 | <.001 |
|  | R Inf. Longitudinal Fasciculus | .495 | .036 | <.001 |
| General WMH | WMH volume | .940 | .023 | <.001 |
|  | Fazekas Deep Total | .694 | .028 | <.001 |
|  | Fazekas Periventricular Total | .757 | .023 | <.001 |

Note: L = left hemisphere, R = right hemisphere.

*Figure S1*. Simplified diagrams of the three alternative model classes tested, based on Kievit et al. (2012; cited in the main article). (a) A simple ‘neuro-*g*’ model where brain and cognition stem from a single factor. (b) A correlational model where a general factor of brain structure, neuro-*g*, is correlated with a general factor of cognitive ability, *g*. (c) A reversal of the MIMIC model shown in Fig. 2, where a general factor of brain structure, neuro-g, is predicted by each of the cognitive tests. See Table S5 for fit statistics for each of the models. All three models included a bifactor structure for the cognitive tests, and a hierarchical structure for the brain variables, as in the original model. Note: Cog. test = Cognitive test; Brain var. = Brain variable (from neuroimaging).

*Table S5*. Fit statistics for the three classes of alternative models (see Fig. S1 for diagrams and description) and the MIMIC models used in the main paper (see Fig. 2).

| Model type | Model | *χ^2^* | df | *p*-value | CFI | TLI | RMSEA | SRMR | saBIC | *r_g-_*_neuro-_*_g_* | *g*/neuro-*g* shared variance |
| --- | --- | --- | --- | --- | --- | --- | --- | --- | --- | --- | --- |
| Single-factor ‘neuro-*g*’ | 1: Overall *g* TBV | 1631.628 | 602 | <.001 | 0.885 | 0.873 | 0.050 | 0.124 | 59688.21 | - | - |
|  | 2: Fluid *g* TBV | 1003.363 | 319 | <.001 | 0.871 | 0.847 | 0.057 | 0.126 | 45863.93 | - | - |
|  | 3: Overall *g* Cortical Split | 1810.997 | 602 | <.001 | 0.868 | 0.854 | 0.055 | 0.124 | 59547.78 | - | - |
|  | 4: Fluid *g* Cortical Split | 1016.897 | 319 | <.001 | 0.872 | 0.848 | 0.057 | 0.127 | 45570.62 | - | - |
| Two-factor correlation | 1: Overall *g* TBV | 1454.717 | 601 | <.001 | 0.905 | 0.894 | 0.046 | 0.093 | 59516.75 | .441 | 19.4% |
|  | 2: Fluid *g* TBV | 971.763 | 331 | <.001 | 0.879 | 0.862 | 0.054 | 0.109 | 45788.72 | .473 | 22.3% |
|  | 3: Overall *g* Cortical Split | 1549.177 | 601 | <.001 | 0.896 | 0.885 | 0.048 | 0.096 | 59295.27 | .496 | 24.6% |
|  | 4: Fluid *g* Cortical Split | 1065.215 | 331 | <.001 | 0.865 | 0.846 | 0.057 | 0.115 | 45575.51 | .497 | 24.7% |
| Reverse MIMIC | 1: Overall *g* TBV | 1178.212 | 596 | <.001 | 0.935 | 0.927 | 0.038 | 0.050 | 59255.24 | - | 25.2% |
|  | 2: Fluid *g* TBV | 629.474 | 366 | <.001 | 0.953 | 0.945 | 0.033 | 0.041 | 48930.04 | - | 20.4% |
|  | 3: Overall *g* Cortical Split | 1240.603 | 596 | <.001 | 0.929 | 0.921 | 0.040 | 0.063 | 59007.28 | - | 17.9% |
|  | 4: Fluid *g* Cortical Split | 634.271 | 366 | <.001 | 0.954 | 0.945 | 0.033 | 0.041 | 48627.10 | - | 19.4% |
| MIMIC | 1: Overall *g* TBV | 1024.01 | 586 | <*.*001 | 0.951 | .945 | 0.033 | 0.043 | 59149.41 | - | 18.4% |
|  | 2: Fluid *g* TBV | 576.58 | 324 | <*.*001 | 0.952 | .945 | 0.034 | 0.041 | 45434.25 | - | 21.1% |
|  | 3: Overall *g* Cortical Split | 1043.69 | 586 | <*.*001 | 0.948 | .942 | 0.034 | 0.044 | 58852.96 | - | 17.9% |
|  | 4: Fluid *g* Cortical Split | 588.12 | 324 | <*.*001 | 0.949 | .941 | 0.035 | 0.042 | 45139.20 | - | 20.9% |

Note: CFI = Comparative Fit Index; TLI = Tucker-Lewis Index; RMSEA = Root Mean Square Error of Approximation; SRMR = Standardized Root Mean Square Residual; saBIC = sample-adjusted Bayesian Information Criterion. TBV = Total Brain Volume.

**Sex Difference Analysis**

In order to investigate whether the key regression parameters differed between males and females, we conducted multi-group SEM analyses. Input data were residualized for participants’ age and handedness. We estimated each of the four MIMIC models. We established metric invariance in the latent constructs, by first estimating the model freely in a joint model (configural invariance) and next constraining the factor loadings to be equivalent across groups (metric invariance). We assessed whether the invariance assumptions were supported by model fit comparisons. We followed the recommendations of Chen (2007), and took a difference in CFI of −0.01 along with a difference in RMSEA of 0.015 as indicative of the invariance assumptions being violated. As can be seen from Table S6, there was no significant decline in model fit when the invariance constraints were placed on the models, indicating metric invariance held across males and females.

*Table S6.* Model fit statistics for multi-group invariance analyses across males and females.

| Model | *χ^2^* | df | *p*-value | CFI | TLI | RMSEA | SRMR |
| --- | --- | --- | --- | --- | --- | --- | --- |
| Overall *g* |  |  |  |  |  |  |  |
| Config | 1698.933 | 1174 | <.001 | 0.943 | 0.936 | 0.036 | 0.053 |
| Metric | 1764.181 | 1223 | <.001 | 0.941 | 0.936 | 0.036 | 0.057 |
| *Δfit* | *65.248* | *49* | *ns* | *−0.002* | *0* | *0* | *0.004* |
|  |  |  |  |  |  |  |  |
| Fluid *g* |  |  |  |  |  |  |  |
| Config | 938.346 | 648 | <.001 | 0.946 | 0.938 | 0.037 | 0.050 |
| Metric | 970.118 | 672 | <.001 | 0.945 | 0.939 | 0.036 | 0.055 |
| *Δfit* | *31.772* | *24* | *ns* | *−0.001* | *0.001* | *−0.001* | *0.005* |

Next, we sequentially placed parameter constraints on each of the regression coefficients in each of the four models. Constraints were added individually. This a Satorra-Bentler adjusted chi-square difference test, with 1 degree of freedom in each case. Specicially, we tested whether our model fit declined significantly when we estimated a single parameter for males and females. A significant difference in fit would indicate the relation between the predictor and the cognitive latent variable differed between males and females. Results are presented in Table S7.

*Table S7.* Difference in model fit for equivalence constraints of regression parameters of interest.

|  | Overall *g* | | | Fluid *g* | | |
| --- | --- | --- | --- | --- | --- | --- |
|  | *SBΔχ^2^* | df | *p*-value | *SBΔχ^2^* | df | *p*-value |
| TBV | 0.034 | 1 | >.05 | 0.241 | 1 | >.05 |
| FA | 0.185 | 1 | >.05 | 0.157 | 1 | >.05 |
| WML | 0.223 | 1 | >.05 | 0.011 | 1 | >.05 |
| Iron Deposits | 2.562 | 1 | >.05 | 0.399 | 1 | >.05 |
| Microbleeds | 0.647 | 1 | >.05 | 1.493 | 1 | >.05 |
| Cortical Thickness | 2.329 | 1 | >.05 | 3.386 | 1 | >.05 |
| Cortical Volume | 2.950 | 1 | >.05 | 0.611 | 1 | >.05 |
| Subcortical Volume | 1.837 | 1 | >.05 | 0.307 | 1 | >.05 |

*Note:* Critical value for *χ^2^*(1) = 3.841

**Supplemental Reference**

Chen, F.F., Sensitivity of goodness of fit indexes to lack of measurement invariance. *Structural Equation Modeling*, **14**, 2007, 464-504, http://dx.doi.org/10.1080/10705510701301834
